# Prider: multiplexed primer design using linearly scaling approximation of set coverage

**DOI:** 10.1186/s12859-022-04710-1

**Published:** 2022-05-12

**Authors:** Niina Smolander, Timothy R. Julian, Manu Tamminen

**Affiliations:** 1 Department of Biology, University of Turku, 20014 Turku, Finland; 2 Department of Environmental Microbiology, Eawag, Swiss Federal Institute of Aquatic Science and Technology, 8600 Dübendorf, Switzerland

**Keywords:** R, C++11, Oligonucleotide primers, Oligonucleotide probes

## Abstract

**Background:**

Designing oligonucleotide primers and probes is one of the key steps of various laboratory experiments such as multiplexed PCR or digital multiplexed ligation assays. When designing multiplexed primers and probes to complex, heterogeneous DNA data sets, an optimization problem can arise where the smallest number of oligonucleotides covering the largest diversity of the input dataset needs to be identified. Tools that provide this optimization in an efficient manner for large input data are currently lacking.

**Results:**

Here we present Prider, an R package for designing primers and probes with a nearly optimal coverage for complex and large sequence sets. Prider initially prepares a full primer coverage of the input sequences, the complexity of which is subsequently reduced by removing components of high redundancy or narrow coverage. The primers from the resulting near-optimal coverage are easily accessible as data frames and their coverage across the input sequences can be visualised as heatmaps using Prider’s plotting function. Prider permits efficient design of primers to large DNA datasets by scaling linearly to increasing sequence data, regardless of the diversity of the dataset.

**Conclusions:**

Prider solves a recalcitrant problem in molecular diagnostics: how to cover a maximal sequence diversity with a minimal number of oligonucleotide primers or probes. The combination of Prider with highly scalable molecular quantification techniques will permit an unprecedented molecular screening capability with immediate applicability in fields such as clinical microbiology, epidemic virus surveillance or antimicrobial resistance surveillance.

**Supplementary Information:**

The online version contains supplementary material available at 10.1186/s12859-022-04710-1.

## Background

Multiplex molecular techniques, such as multiplex polymerase chain reaction [1] and digital multiplex ligation assay (dMLA) [2], are methods for detecting and quantifying multiple genomic targets in a single experiment. These techniques have enabled the development of various screening methods in the fields of pathogen detection and human genetics and utilise sets of primers or probes that can detect hundreds of targets [3–7].

Designing primers or probes for optimal detection of multiple targets in complex and large sets of DNA sequences is a set coverage problem which aims to find a minimal set of primer sequences that cover the input DNA sequences [8]. Various tools have been created for multiplex primer and probe designing, such as the command line based *PriMux* [9], the web-application *PrimerDesign* [10], the R package *DECIPHER*’s *DesignPrimers* and *DesignProbes* [11], the GUI *PrimerMapper* [12] and the R package *openPrimeR* [13]. However, most of these tools no longer appear to be available or functional and/or require significant user intervention via requiring an external options file for the parameters or a file conversion from a FASTA file and/or scale poorly to large input data. The key features of these tools are compared with Prider in the Additional file 1.

Here we present an R package Prider, which computes a near-optimal primer coverage for input FASTA file and scales linearly to increasing sequence data. Prider is a flexible tool which permits designing primers and probes for highly scalable molecular screening and quantification applications [2–5]. The key features of Prider are its suitability for scripting, capability of approximating near-optimal set coverage with minimal user intervention, linear scalability to increasing data, and inbuilt capability to visualise the estimated coverage. These features improve the scalability of multiplex molecular techniques and have immediate applicability in fields such as clinical microbiology, epidemic virus surveillance or antimicrobial resistance surveillance.

## Implementation

### Input and parameters

Prider was developed on R version 4.0.5 [14] with the package Rcpp 1.0.7 [15] using C++11. The input to Prider is a single FASTA file containing the sequences to which primers/probes are to be designed. Users can change the primer length, the minimum primer and sequence group sizes and the number of cumulative coverage decimals, explained below. Furthermore, optional filtering removes the primers with proportional G and C base contents outside the user-specified range. Another optional filtering removes the primers exceeding a user-defined difference in proportional GC content between the two halves of the primer. This filtering is aimed primarily for designing adjacent probes that during Prider processing are considered to be one oligonucleotide.

### Cluster preparation and filtering

The first step of primer cluster preparation is the division of each DNA sequence from the input FASTA file into sub-sequences—primer candidates—of user-specified length using a sliding window function. During the process, the primer candidates remain associated with their respective FASTA headers. Subsequently, primer candidates shared by multiple input sequences are used to group together sequences with shared motifs. These sequence groups are further grouped together, linking different primer candidates together and producing a data frame of all sequence clusters and primer clusters which cover them.

To optimize the number of primer candidates needed to cover the input FASTA, the primer clusters with target sequence coverage or sequence cluster size below the user-defined cut-offs are excluded. The primer clusters are subsequently ordered by their size, and the cumulative contributions of each cluster to the total sequence coverage are calculated and rounded based on a user-defined value. Finally, primer clusters with the same cumulative coverage are grouped together and only the clusters with the largest sequence and primer group sizes are kept. This step reduces the number of primer clusters that share equal or very similar sequence coverage.

### Prider output

The output of Prider is an S3-decorated list with five elements accessible with Prider’s S3 methods, indexing, or the “$” operator. Detailed functionality of the S3 methods is explained in the reference manual at https://CRAN.R-project.org/package=prider. The output elements are:

1. Description; summarises the contents of the input FASTA and the produced Prider list.
2. Conversion table; a data frame containing the original FASTA headers, full DNA sequences and the sequence ids.
3. Primer candidates; a data frame containing the primer group DNA sequences, an identification number for each primer group, the sequence ids associated with the primer clusters, primer cluster and sequence group sizes and the cumulative coverage values.
4. Excluded sequences; a data frame containing the sequences not associated with any primer cluster due to filtering criteria.
5. Primer matrix; a TRUE–FALSE table where each row is a primer group and each column a single sequence id. This is the input for the S3 plotting function for the Prider objects.

**Fig. 1:**
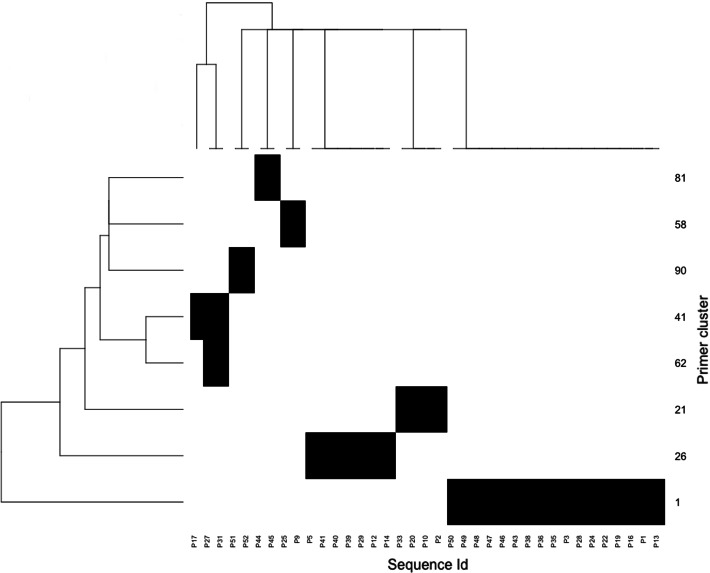
Heatmap of the distribution of the primer clusters designed for the example FASTA file. Each Sequence Id represents a sequence in the input FASTA file and each Primer cluster represents a set of primers designed by Prider. Black areas of the heatmap indicate coverage of Sequence Id(s) by a Primer cluster

Prider provides S3 methods primers and sequences to access the primer clusters and their sequence coverage, respectively, and a method for plotting (Fig. 1).

## Results and discussion

Processing speed of Prider was evaluated using two randomly generated FASTA file sets; one with increasing number of bases per file (300 sequences each) and one with increasing number of sequences per file (465,000 bases each). The sets consisted of 310 and 300 files, respectively, and 10 replicates of each number of bases or sequences. To make sure that even the smallest files could be processed, the parameter *minimum_sequence_group_size* was set to 1. Similar test with a subset of the FASTA file set with increasing number of bases per file was performed with the R package openPrimeR. No other tools were tested due to reasons listed in Additional file 1.

**Fig. 2:**
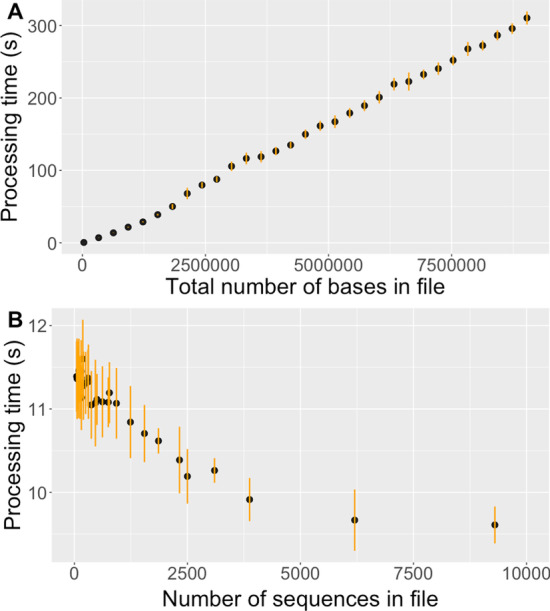
Prider processing time (dot) and standard deviation (line) for **A** varying numbers of bases in the benchmarking files (each file containing 300 sequences), **B** varying numbers of sequences in the benchmarking files (each file containing 465,000 bases)

The processing time of Prider, determined by the *user.self* value of the base R function *system.time*, was linearly dependent on the number of input bases, with 3e4 bases taking approx. 0.5 s and 9.03e6 bases taking approx. 310 s (Fig. 2A) on a Macbook Pro (M1, 8 GB, 2020, macOS Big Sur). The number of sequences the bases were distributed on had a minor, decreasing effect on the processing time (Fig. 2B). The test data and the code used for the tests are available at Zenodo (https://zenodo.org/record/6483171#.YmaiEvNBxAc). The full benchmarking results are available as Additional files 2 and 3. The comparison of the processing speeds of Prider and openPrimeR shows that Prider processes files many times faster than openPrimeR. Full comparison is available as an Additional file 4. The benchmarks reveal that Prider scales well to large sequence data and has low variation between the processing times of the replicates.

## Conclusions

Design of multiplexed primers and probes to highly diverse DNA data is a problem commonly encountered in various screening applications [2–5]. For instance, in pathogenicity detection, clinical virology and antimicrobial resistance surveillance one needs to account for the extremely high diversity of relevant genes [16–18]. Such screening applications greatly benefit from Prider since its linear scalability allows for the processing of large and complex sequence data required for comprehensive probe design. Thus, combination of Prider with highly scalable molecular quantification techniques such as dMLA will permit an unprecedented molecular screening capability with immediate applicability in fields such as clinical microbiology, epidemic virus surveillance or antimicrobial resistance surveillance.

### Availability and requirements

- Project name: Prider.
- Project home page: https://github.com/tamminenlab/prider; https://CRAN.R-project.org/package=prider
- Operating systems: Platform independent.
- Programming languages: R, C++11.
- Other requirements: R version ≥ 4.0.0, C++11.
- License: BSD 3 clause.
- Any restrictions to use by non-academics: None

## Supplementary Information

**Additional file 1: **Comparison of multiplex primer and probe designing tools. The table compares the following features of the multiplex primer/probe designing tools *PriMux*, *PrimerDesign*, *DECIPHER*, *PrimerMapper*, *openPrimeR* and Prider: the type of tool, project status, the required sequence input file format, and if external dependencies are required. The table also contains notes and a link for each tool**.**

**Additional file 2: **Table of Prider benchmark test files’ metadata and *system.time* output for the increasing number of nucleotides dataset. The data includes the number of sequences, the number of bases, the mean number of bases per sequence and the base standard deviation as well as the *system.time* function output values *user.self*, *sys.self*, *elapsed*, *user.child* and *sys.child* of each Prider processed FASTA file for the increasing number of nucleotides dataset**.**

**Additional file 3: **Table of Prider benchmark test files’ metadata and *system.time* output for the increasing number of sequences dataset. The data includes the number of sequences, the number of bases, the mean number of bases per sequence and the base standard deviation as well as the *system.time* function output values *user.self*, *sys.self*, *elapsed*, *user.child* and *sys.child* for each Prider processed FASTA file of the increasing number of sequences dataset**.**

**Additional file 4: **Table of Prider and openPrimeR benchmark test files’ metadata and *system.time* output for the increasing number of nucleotides dataset. The data includes the number of sequences, the number of bases, the mean number of bases per sequence and the base standard deviation as well as the *system.time* function output values *user.self*, *sys.self*, *elapsed*, *user.child* and *sys.child* of each openPrimeR and Prider processed FASTA file for the increasing number of nucleotides dataset**.**

## Data Availability

Prider is available from GitHub as an R package (https://github.com/tamminenlab/prider) and from CRAN (https://CRAN.R-project.org/package=prider). The version referenced in this article is available from Zenodo (https://doi.org/10.5281/zenodo.5713605). The datasets generated and analysed during the current study are available in the Zenodo repository, https://zenodo.org/record/6483171#.YmaiEvNBxAc. The datasets supporting the conclusions of this article are included within the article and its additional files.

## Abbreviation

dMLA: Digital multiplex ligation assay
